# Synthesis and preclinical evaluation of novel ^18^F-vancomycin-based tracers for the detection of bacterial infections using positron emission tomography

**DOI:** 10.1007/s00259-024-06717-7

**Published:** 2024-04-22

**Authors:** G. B. Spoelstra, S. N. Blok, L. Reali Nazario, L. Noord, Y. Fu, N. A. Simeth, F. F. A. IJpma, M. van Oosten, J. M. van Dijl, B. L. Feringa, W. Szymanski, P. H. Elsinga

**Affiliations:** 1 Department of Nuclear Medicine and Molecular Imaging, University of Groningen, University Medical Center Groningen, Hanzeplein 1, Groningen, 9713GZ The Netherlands; 2 Department of Trauma Surgery, University of Groningen, University Medical Center Groningen, Hanzeplein 1, Groningen, 9713GZ The Netherlands; 3 Stratingh Institute for Chemistry, University of Groningen, Nijenborgh 7, Groningen, 9747AG The Netherlands; 4 Institute for Organic and Biomolecular Chemistry, Department of Chemistry, University of Göttingen, Tammannstraβe 2, 37077 Göttingen, Germany; 5 Department of Medical Microbiology and Infection Prevention, University of Groningen, University Medical Center Groningen, Hanzeplein 1, Groningen, 9713GZ The Netherlands; 6 Department of Radiology, University of Groningen, University Medical Center Groningen, Hanzeplein 1, Groningen, 9713GZ The Netherlands; 7 Department of Medicinal Chemistry, Photopharmacology and Imaging, University of Groningen, Groningen Research Institute of Pharmacy, Antonius Deusinglaan 1, Groningen, 9713AV The Netherlands

**Keywords:** Positron emission tomography, Fluorine-18, Bacterial infection imaging, Biodistribution, Gram-positive, Vancomycin

## Abstract

**Introduction:**

Bacterial infections are a major problem in medicine, and the rapid and accurate detection of such infections is essential for optimal patient outcome. Bacterial infections can be diagnosed by nuclear imaging, but most currently available modalities are unable to discriminate infection from sterile inflammation. Bacteria-targeted positron emission tomography (PET) tracers have the potential to overcome this hurdle. In the present study, we compared three ^18^F-labelled PET tracers based on the clinically applied antibiotic vancomycin for targeted imaging of Gram-positive bacteria.

**Methods:**

[^18^F]FB-NHS and [^18^F]BODIPY-FL-NHS were conjugated to vancomycin. The resulting conjugates, together with our previously developed [^18^F]PQ-VE1-vancomycin, were tested for stability, lipophilicity, selective binding to Gram-positive bacteria, antimicrobial activity and biodistribution. For the first time, the pharmacokinetic properties of all three tracers were compared in healthy animals to identify potential binding sites.

**Results:**

[^18^F]FB-vancomycin, [^18^F]BODIPY-FL-vancomycin, and [^18^F]PQ-VE1-vancomycin were successfully synthesized with radiochemical yields of 11.7%, 2.6%, and 0.8%, respectively. [^18^F]FB-vancomycin exhibited poor in vitro and in vivo stability and, accordingly, no bacterial binding. In contrast, [^18^F]BODIPY-FL-vancomycin and [^18^F]PQ-VE1-vancomycin showed strong and specific binding to Gram-positive bacteria, including methicillin-resistant *Staphylococcus aureus* (MRSA), which was outcompeted by unlabeled vancomycin only at concentrations exceeding clinically relevant vancomycin blood levels. Biodistribution showed renal clearance of [^18^F]PQ-VE1-vancomycin and [^18^F]BODIPY-FL-vancomycin with low non-specific accumulation in muscles, fat and bones.

**Conclusion:**

Here we present the synthesis and first evaluation of the vancomycin-based PET tracers [^18^F]BODIPY-FL-vancomycin and [^18^F]PQ-VE1-vancomycin for image-guided detection of Gram-positive bacteria. Our study paves the way towards real-time bacteria-targeted diagnosis of soft tissue and implant-associated infections that are oftentimes caused by Gram-positive bacteria, even after prophylactic treatment with vancomycin.

**Supplementary Information:**

The online version contains supplementary material available at 10.1007/s00259-024-06717-7.

## Introduction

Bacterial infections are a major problem in medicine, and they impose an enormous economic burden on society. It is estimated that, in Europe alone, 4.1 million patients suffer from 8.9 million healthcare-associated infections (HAIs) each year [1]. A steadily ageing population and advances in surgery have led to an increase in elective surgeries with the consequence of increased numbers of surgical site infections [2, 3]. This is clearly evident in orthopedic and trauma surgery, where joints are reconstructed, or fractures stabilized with the aid of foreign body materials. The resulting prosthetic joint infections (PJIs) and fracture-related infections (FRIs) are notoriously difficult to diagnose, and their treatment is difficult [4–8]. Late diagnosis or ineffective treatment of PJIs and FRIs may lead to persistent infections that severely affect the patients’ wellbeing due to repeated surgery, loss of limbs or even mortality [9, 10]. The formation of biofilms on implanted devices, such as prosthetic joints and osteosynthesis materials, further complicates diagnosis and treatment [11]. Such biofilms may emerge within weeks after surgery, but they may also develop gradually in the course of months or years. The symptoms of biofilm-associated infections range from acute and fulminant inflammation to low-grade, but biofilms may also persist asymptomatically [12]. Unfortunately, the bacteria inside matured biofilms are highly refractive to antimicrobials, making successful treatment of the infection with antibiotics close to impossible [13–15].

The gold standard in diagnosis of bacterial infections remains the collection and culturing of samples, which requires invasive and sometimes risky sampling techniques, whereas the results may be confounded by contamination with the patient’s microbiota [16, 17]. Furthermore, sample workup is slow, hampering rapid clinical decision making in critical situations [18, 19], and the oftentimes polymicrobial nature of biofilms may result in an incomplete or false diagnose [17, 20]. Yet, to ensure optimal treatment of patients with a suspected bacterial infection, and to mitigate unnecessary or sub-optimal medical interventions, it is of decisive importance that the diagnosis is rapid and accurate. Radiological imaging modalities, such as Computed Tomography (CT), Magnetic Resonance Imaging (MRI) and X-ray imaging, or ultrasound scanning, can aid in the diagnosis of infections, but they are of little use when anatomical defects are not clearly evident. Also, such modalities cannot distinguish between inflammation due to infection or due to a foreign body reaction [15, 21, 22]. This diagnostic ‘valley of death’ can, however, be bridged by molecular imaging through the application of bacteria-targeted near-infrared (NIR) fluorescent tracers or, even more promising, bacteria-targeted tracers that can be tracked by positron emission tomography (PET).

Nuclear medicine is emerging as a key modality in bacterial infection imaging that combines high sensitivity and specificity [21, 23–27]. Today, this mostly involves 2-deoxy-2-[^18^F]fluoro-d-glucose ([^18^F]FDG) PET/CT or radiolabeled white blood cell scintigraphy (WBC SPECT) [28]. [^18^F]FDG is usually applied to identify the cause of fevers with unknown origin [29]. However, [^18^F]FDG is taken up by all metabolically active cells, without discriminating between malignant cells and invasive pathogens, or inflamed and healthy tissue [30]. WBC SPECT, on the other hand, requires the collection and labeling of white blood cells from a patient, and their subsequent re-administration [31, 32]. Importantly, as both [^18^F]FDG PET and WBC SPECT predominantly visualize inflammation rather than the presence of invasive bacteria, bacteria-specific radio-labelled tracers are needed to diagnose bacterial infections by PET [33]. This requirement could be met by [^18^F]2-fluoro-2-deoxy-sorbitol ([^18^F]FDS) [34, 35], but this requires uptake of the [^18^F]FDS which occurs mostly in Gram-negative bacteria that are not commonly associated with PJIs or FRIs. Instead, most of these implant-associated infections are caused by Gram-positive bacteria [13].

As an alternative to tracers based on metabolites, fluorescently or radio-labelled tracers based on antimicrobials, such as trimethoprim [36], ciprofloxacin [37] and vancomycin [38, 39] have shown great promise to specifically detect bacteria by molecular imaging. Vancomycin, a glycopeptide antibiotic that selectively targets d-Ala-d-Ala moieties in the bacterial cell wall, is widely used to combat Gram-positive bacterial infections [40]. Importantly, previous preclinical studies have shown that a conjugate of vancomycin and the NIR fluorophore IRDye800CW (vanco-800CW) allows the selective detection of Gram-positive bacterial infections in vivo [41–43]. These promising results with vanco-800CW inspired us to also develop vancomycin-based PET tracers, because the emitted gamma radiation of PET tracers would allow the detection of deep-seated infections throughout the human body, which is not possible with a NIR tracer [44]. For this purpose, we considered fluorine-18 as radionuclide, because it has a relatively short half-life (109.8 min) and can be produced in large quantities.

Here we describe our study aimed at the design, development, in vitro specificity testing, and in vivo biodistribution testing of three PET tracers based on vancomycin, [^18^F]FB-vancomycin, [^18^F]BODIPY-FL-vancomycin and [^18^F]PQ-VE1-vancomycin, towards selective imaging of Gram-positive bacterial infections.

## Materials & Methods

### Reagents

Vancomycin was obtained from the Pharmacy department of the University Medical Center Groningen (UMCG, Groningen, The Netherlands) in 1 g vials (vancomycin Hikma, 1000 mg for infusion). Precursor for [^18^F]SFB (4-(ethoxycarbonyl)-*N*,*N*,*N*-trimethylbenzenaminium triflate) and reference standard [^19^F]SFB were purchased from ABX (Radeberg, Germany), [^19^F]BODIPY-FL-NHS from Lumi-Probe (Hannover, Germany), [^19^F]BODIPY-FL-vancomycin conjugate from Thermo Fisher Scientific (Waltham, United States) and tin(IV)chloride from Sigma-Aldrich (Darmstadt, Germany).

### General culturing conditions

Bacterial strains were cultured in Tryptic Soy Broth (TSB) at 37 °C and 250 revolutions per min (rpm) in an orbital shaker (Forma Scientific 4520, Marietta, United States). Prior to culturing in TSB, staphylococci were grown on Tryptic Soy Agar (TSA) and *Escherichia coli* (*E. coli*) on Lysogeny Broth (LB) agar overnight at 37 °C as indicated. To determine colony-forming units (CFUs), the bacteria were plated on Columbia III agar (Becton Dickinson, Eysins, Switzerland). The bacterial strains used in this study were clinical isolates of *Cutibacterium acnes* (*C. acnes*), *E. coli*, *Enterobacter cloacae* (*E. cloacae*), *Staphylococcus capitis* (*S. capitis*), and clinical isolates of *Staphylococcus aureus* (*S. aureus*) that had been collected at the University Medical Center Groningen in October 2015 and February 2024, *Enterococcus faecalis* (*E. faecalis*) ATCC 29,212 [45], *Klebsiella pneumoniae* (*K. pneumoniae*) ATCC 700,603 [46], *Pseudomonas aeruginosa* (*P. aeruginosa*) ATCC 27,853 [47], the methicillin-resistant *S. aureus* (MRSA) USA300 [48], *S. epidermidis* ATCC 35,984 [49] and *S. epidermidis* ATCC 12,228 [50]. The strains were stored as glycerol stocks at -80 °C.

### Radiochemistry

#### Fluorine-18 production and preparation

[^18^F]Fluoride was produced by irradiation of [^18^O]H_2_O using an IBA Cyclone 18/9 Twin (Ion Beam Applications, Louvain-la-Neuve, Belgium) equipped with a conical-5 target via the ^18^O(p, n)^18^F nuclear reaction. A Sep-Pak Light Accell Plus QMA anion exchange cartridge (Waters, Milfort, United States) was preconditioned with 5 mL NaHCO_3_ and 10 mL H_2_O and used to trap the [^18^F]fluoride. In a typical synthesis, 20 GBq [^18^F]fluoride was used for the synthesis of [^18^F]FB-vancomycin, [^18^F]BODIPY-FL-vancomycin or [^18^F]PQ-VE1-vancomycin.

#### Synthesis of [^18^F]FB-vancomycin

##### Radiosynthesis of N-succinimidyl 4-[^18^F]fluorobenzoate

The synthesis of *N*-succinimidyl 4-[^18^F]fluorobenzoate ([^18^F]SFB) was performed as previously described [51]. Briefly, an IBA Synthera synthesis module was equipped with a PC120 cassette (Ion Beam Applications, Louvain-la-Neuve, Belgium). In preparation of the synthesis, 5.0 mg SFB-precursor was azeotropically dried under nitrogen with three additions of anhydrous acetonitrile (MeCN) prior to dissolving in anhydrous DMSO. The trapped [^18^F]fluoride was eluted using 20 mg Kryptofix_222_ and 3.5 mg K_2_CO_3_ in a mixture of 700 µL of MeCN and 200 µL of H_2_O. The Kryptofix complex was dried at 110 °C for 5 min, followed by three additions of 0.5 mL anhydrous MeCN under vacuum and nitrogen flow. After drying, the [^18^F]SFB-precursor in anhydrous DMSO was added to the reactor. ^18^F-Fluorination was performed at 110 °C for 15 min. For ester hydrolysis, 20 µL tetramethylammonium hydroxide (1 M, Sigma-Aldrich Darmstadt, Germany) in anhydrous MeCN was added and the solution was heated to 95 °C for 15 min under vacuum and argon flow. The 4-[^18^F]fluorobenzoate was converted to [^18^F]SFB by the addition of 20 mg *N*,*N*,*N*’,*N*’-tetramethyl-*O*-(*N*-succinimidyl)uronium tetrafluoroborate (TSTU, Sigma-Aldrich, Darmstadt, Germany) in 1 mL of anhydrous MeCN and heating to 110 °C for 5 min to yield [^18^F]SFB. [^18^F]SFB was transferred to a water vial containing 60 mL of water for formulation. The formulation was performed using an Oasis HLB 1 cc (30 mg) cartridge. After washing the cartridge with 10 mL water, the product was eluted from the cartridge using 1.2 mL 100% EtOH.

##### Conjugation of [^18^F]SFB to Vancomycin

[^18^F]SFB was conjugated to vancomycin using standard NHS-mediated amidation conditions. Vancomycin (5.0 mg) was dissolved in 1.0 mL sodium borate in water (100 mM, pH 8.4). [^18^F]SFB in 1.2 mL 100% EtOH was added, and the mixture was allowed to react for 10 min at room temperature. After conjugation, 2.0 mL of the reaction mixture was injected on HPLC and the product peak was collected at 9 min (column: Waters xBridge BEH Shield RP18 130 Å, 3.5 μm, solvent 75% of 0.1% TFA in water / 25% MeCN, flow: 5 mL · min^− 1^). Product was added to 60 mL water and transferred over an Oasis HLB 1 cc (30 mg) cartridge. After washing the cartridge with 10 mL water, the product was eluted from the cartridge using 500 µL 100% EtOH, after which the EtOH concentration was brought below 5% using 0.9% saline solution for subsequent experiments.

#### Synthesis of [^18^F]BODIPY-FL-vancomycin

##### Radiosynthesis of [^18^F]BODIPY-FL-NHS

For the radiolabeling of [^18^F]BODIPY-FL-NHS, an isotope exchange reaction (IEX) was used as described in [52]. The trapped [^18^F]fluoride was eluted using 3.5 mg tetraethylammonium bicarbonate in 1 mL MeOH. The [^18^F]fluoride was azeotropically dried at 130 °C under nitrogen flow, followed by three additions of 0.5 mL anhydrous MeCN. When dry, 1.0 mL anhydrous MeCN containing 20 µL tin(IV)chloride was added to 200 µg [^19^F]BODIPY-FL-NHS and subsequently added to the anhydrous [^18^F]fluoride complex. While stirring, it was allowed to react for 10 min at room temperature. The reaction mixture was added to 60 mL of water and subsequently transferred over an Oasis HLB 3 cc (60 mg) cartridge. The cartridge was washed with 10 mL of water prior to elution to remove all unreacted [^18/19^F]fluoride. Next, the product was eluted from the cartridge using 1 mL 100% EtOH.

##### Conjugation of [^18^F]BODIPY-FL-NHS to Vancomycin

[^18^F]BODIPY-FL-NHS in 100% EtOH (1 mL) was added to 5.0 mg vancomycin in 1 mL NaHCO_3_ (100 mM, pH 8.4) and stirred at 35 °C for 20 min. Next, the reaction mixture was injected on HPLC, and the product peak was collected at 7 min (column: xBridge BEH Shield RP18 130 Å, 3.5 μm, solvent 70% of 0.1% TFA in water / 30% MeCN, flow: 5 mL · min^− 1^). Product was added to 60 mL water and transferred over an Oasis HLB 1 cc (30 mg) cartridge. After washing the cartridge with 10 mL water, the product was eluted from the cartridge using 500 µL 100% EtOH, after which the EtOH concentration was brought below 5% using 0.9% saline solution for subsequent experiments.

#### Radiosynthesis of [^18^F]PQ-VE1-vancomycin

[^18^F]PQ-VE1-vancomycin was synthesized in a batch LED-reactor as previously described in [44]. Starting materials, i.e. PQ-vancomycin and VE1-tosylate, were synthesized as previously detailed in the Supporting Info of reference [44]. Briefly, all reagents and equipment were dried and purged of oxygen using nitrogen gas. The trapped [^18^F]fluoride was eluted using 15.0 mg Kryptofix_222_ and 1.0 mg KHCO_3_ in a mixture of 700 µL of MeCN and 200 µL of water. The [^18^F]fluoride was azeotropically dried at 130 °C with three additions of anhydrous, degassed, MeCN. When dry, 3.0 mg vinyl ether tosylate (VE1-tos) in 500 µL anhydrous MeCN was added and heated to 110 °C for 3 min in a sealed 5 mL conical vial, to minimize the ingress of water and oxygen, and to minimize the loss of volatile ^18^F-fluorinated vinyl-ether intermediate. Next, the ^18^F-fluorinated vinyl-ether was distilled into the photoreactor under nitrogen flow. The photoreactor, consisting of a 2 mL vented borosilicate vial over a single 10 W LED cob (emission peak at 395 nm), was preloaded with 3.0 mg PQ-vancomycin dissolved in 500 µL degassed H_2_O/MeCN (60/40 v/v). The photoreactor was activated for 300 s to irradiate the [^18^F]VE1 and PQ-vancomycin mixture, after which the reaction mixture was drawn up in a syringe and water was added for a total volume of 2.0 mL. The mixture was injected on HPLC, and product was collected at 13.2 min (column: xBridge BEH Shield RP18 130 Å, 3.5 μm, solvent A: 0.1% TFA in water, solvent B: MeCN, gradient 0 min: 80% A, 5 min: 80% A, 10 min: 60% A, 30 min: 15% A, flow: 4 mL · min^− 1^). Product was added to 60 mL water and transferred over an Oasis HLB 1 cc (30 mg) cartridge. After washing the cartridge with 10 mL water, the product was eluted from the cartridge using 500 µL 100% EtOH, after which the EtOH concentration was brought below 5% using 0.9% saline solution for subsequent experiments.

### Radiotracer stability

Stability of the tracers was determined in PBS and human plasma. In a typical experiment, 1 MBq of radioactivity (10–20 µL) was added to 250 µL of PBS or human plasma. Samples were heated to 37 °C on a shaker and sampled at fixed intervals. For samples containing human plasma, protein was precipitated using two volumes of cold MeCN. Precipitated samples were briefly centrifuged to pellet the insoluble fraction and supernatant was used for stability assessment. Supernatant (1 µL) was loaded on a TLC plate and imaged using BAS-IP MS 2025 E plates (Fujifilm, Tokyo, Japan) on an Amersham Typhoon Biomolecular Imager (GE Healthcare Bio-Sciences Corp, Chicago, United States) equipped with a phosphor imaging stage. [^18^F]BODIPY-FL-vancomycin has an R_f_ of 0.68 (petroleum ether/MeOH 3:1), [^18^F]FB-vancomycin has an R_f_ of 0.66 (2 M NaOAc/MeOH 5:1). For [^18^F]PQ-VE1-vancomycin, conditions described in [44] were used.

### Distribution coefficient LogD_7.4_

The tracer distribution coefficient in *n*-octanol/PBS pH 7.4 (LogD_7.4_) was determined using a shake-flask methodology. To an Eppendorf tube containing 500 µL *n*-octanol and 500 µL PBS, 10 µL tracer solution in saline was added. The vial was vortexed for 30 s and placed on an Eppendorf shaker for 15 min. After incubation, layers were separated by centrifugation (1 min at 10.000 rpm) and a fraction of the *n*-octanol layer was pipetted off. To minimize contamination of the bottom layer with the *n*-octanol layer, the bottom PBS layer was collected by puncturing the bottom of the Eppendorf tube with a 22G needle and aspirating a fraction of the PBS layer. Collected fractions were weighted to correct for collected volume and subsequently measured on a calibrated gamma counter and corrected for decay. LogD_7.4_ was calculated as ${log}\left(\frac{{counts}_{n-octanol}}{{counts}_{PBS}}\right)$.

### In vitro tracer binding to *S. aureus, E. coli* and other clinically relevant bacteria

Single colonies of clinical *S. aureus* and *E. coli* isolates were used to inoculate TSB and incubated overnight. From the overnight cultures, fresh cultures were started and grown to an optical density at 600 nm (OD_600_) of approximately 3.0. Both strains were incubated with tracer (3.5 MBq in 1 mL 0.9% saline) in a water bath at 37 °C. The final tracer concentration was 120 kBq ± 28 (38.6 pg ± 9.1 and 0.5 pg ± 0.1 of [^18^F]BODIPY-FL-vancomycin and [^18^F]PQ-VE1-vancomycin, respectively) per mL culture. At fixed time intervals (0, 15 and 30 min), aliquots were collected (2.0 mL in a 2 mL Eppendorf tube) and washed twice with sterile PBS (1.0 mL per washing step). In each washing step, the bacteria were pelleted by centrifugation (10.000 rpm, 60 s) and resuspended in fresh PBS. After the last washing step, the bound radioactivity was quantified using a calibrated gamma counter (Wizard2, Perkin Elmer, Waltham, United States). Measurements were corrected for background and decay. Results are reported as counts per s (Bq). To correlate bound tracer with the number of bacteria in a sample, the bacterial suspensions (2 ×) were plated and CFUs were counted.

Clinical isolates of *E. cloacae*, *S. capitis*, *S. aureus*, as well as type strains of *E. coli*, *E. faecalis*, *K. pneumoniae*, *P. aeruginosa* and *S. epidermidis* were grown under aerobic conditions in brain heart infusion broth. A clinical isolate of the anaerobic bacterium *C. acnes* was grown on plate in an anaerobic chamber, and subsequently cultured in liquid medium with minimal headspace. Samples from fresh bacterial cultures were normalized to an OD_600_ of 1.0, fixed in PBS with 0.5% paraformaldehyde (PFA) for 10 min at room temperature, and resuspended in 1 mL sterile PBS. The fixated bacteria were incubated with tracer (133 kBq per mL, 42.8 pg and 0.6 pg of [^18^F]BODIPY-FL-vancomycin and [^18^F]PQ-VE1-vancomycin, respectively) and washed twice with sterile PBS. The remaining radioactivity was quantified using a calibrated gamma counter (Wizard2, Perkin Elmer, Waltham, United States). Measurements were corrected for background and decay. Results are reported as percentage of the total tracer added.

### Competition with unlabeled vancomycin

To determine the competitive binding between unlabeled vancomycin and [^18^F]BODIPY-FL-vancomycin, or unlabeled vancomycin and [^18^F]PQ-VE1-vancomycin, the *S. aureus* USA300 MRSA strain was cultured as described above. [^18^F]FB-vancomycin was excluded due to its limited in vitro and in vivo stability. On the day of the experiment, a fresh culture was started in 10 mL of TSB and grown to an OD_600_ of 2.0. Unconjugated vancomycin was dissolved in sterile PBS and serially diluted to yield final concentrations between 0 and 1024 µg per mL. Bacteria were added to the dilutions to obtain a final OD_600_ of 0.2 and the vial was briefly vortexed to ensure proper mixing. Tracer was added (125 kBq per mL, 40.3 pg and 0.5 pg of [^18^F]BODIPY-FL-vancomycin and [^18^F]PQ-VE1-vancomycin, respectively) and the vial was vortexed again. After 30 min incubation, aliquots were collected in 2 mL Eppendorf tubes and washed twice with PBS by centrifugation. After the last washing step, the bound radioactivity was quantified using a calibrated gamma counter (Wizard2, Perkin Elmer, Waltham, United States). Measurements were corrected for background and decay. Results are reported as 10^3^ counts per s (kBq).

### Minimum Inhibitory Concentration

To compare the Minimum Inhibitory Concentration (MIC) values for BODIPY-FL-vancomycin, PQ-VE1-vancomycin and unlabeled vancomycin, *S. epidermidis* ATCC 35,984 was grown overnight on Mueller Hinton Agar (MHA). FB-vancomycin was excluded due to limited in vitro and in vivo stability. From a single colony an overnight culture was inoculated in Mueller Hinton Broth (MHB) and incubated overnight. On the day of the experiment, a fresh culture was started in 10 mL of MHB until exponential phase was reached. From this, the culture was diluted to an OD_600_ of 0.01 for the MIC experiment. Vancomycin, BODIPY-FL-vancomycin, and PQ-VE1-vancomycin were dissolved in PBS and serially diluted in a 96 well plate, after which the bacteria were added. Growth was recorded using a BioTeK Synergy2 microplate spectrophotometer (Agilent, Santa Clara, United States). The MIC was defined as the compound concentration at which no growth was detectable after 17 h of incubation.

### Biodistribution in vivo

All murine experiments were approved by the Animal Care and Use Committee of the University of Groningen under license protocol number 2114768-01-001. In vivo biodistribution was performed in male C57BL/6 mice (age 8 weeks, 25 g). Per tracer, the animals were divided in three groups based on the tracer distribution times (30 min, 60 min, and 90 min). A total of 27 mice were allocated for the biodistribution experiments, of which 24 were used. Each experimental arm (i.e. time point) consisted of three animals. Due to rapid in vivo degradation of [^18^F]FB-vancomycin, the 90-min PET scan and biodistribution measurement were not performed with this tracer (Supplemental Table 1). Tracer was administered at t = 0 via penile vein injection (2.0 ± 0.9 MBq in 100 µL 0.9% saline solution, Supplemental Table 2). After tracer administration, the animals were transferred to a Siemens Focus 220 microPET small animal scanner (Siemens, Munich, Germany) and scanned for the designated time, followed by a transmission scan of 10 min for attenuation correction. PET data were normalized and corrected for decay, after which the PET data were reconstructed using OSEM2D-Z1-SC-256. The PET data frames used for reconstruction were: 6 × 10 s, 4 × 30 s, 2 × 60 s, 120 s, 180 s, 4 × 300 s, 3 × 600 s, 1200 s or until the designated scan time was reached, taking into account a 10-min transmission scan following the emission scan. Next, animals were terminated, and organs harvested to assay biodistribution in a calibrated gamma counter (Wizard2, Perkin Elmer, Waltham, United States).

### Statistics

Quantitative data are expressed as mean ± SD unless otherwise stated. Statistical analyses were performed by Students t-tests using RStudio (version 2023.06.1). A *p*-value of < 0.05 was considered significant.

## Results

### Chemical synthesis and tracer structures

All chemical syntheses were performed on an Eckert & Ziegler Modular-Lab PharmTracer synthesis module (Supplemental Fig. 1). Following the radiolabelling, [^18^F]PQ-VE1-vancomycin, [^18^F]FB-vancomycin and [^18^F]BODIPY-FL-vancomycin were isolated using HPLC (Supplemental Figs. 2 & 3). Information regarding synthesis time, radiochemical yield, molar activity, and radiochemical purity can be found in Table 1.

**Table 1:** Overview of overall synthesis time, radiochemical yield, and radiochemical purity

Tracer	Overall Synthesis time (min)	Radiochemical Yield (%)	Molar Activity (GBq/µmol)	Radiochemical Purity (%)
[^18^F]FB-vancomycin	109 ± 13	11.7 ± 0.2	28.6 ± 14.5	> 95
[^18^F]BODIPY-FL-vancomycin	96 ± 5	0.8 ± 0.5	5.35 ± 3.91	> 95
[^18^F]PQ-VE1-vancomycin	83 ± 7	2.6 ± 0.7	415 ± 210	> 95

**Fig. 1:**
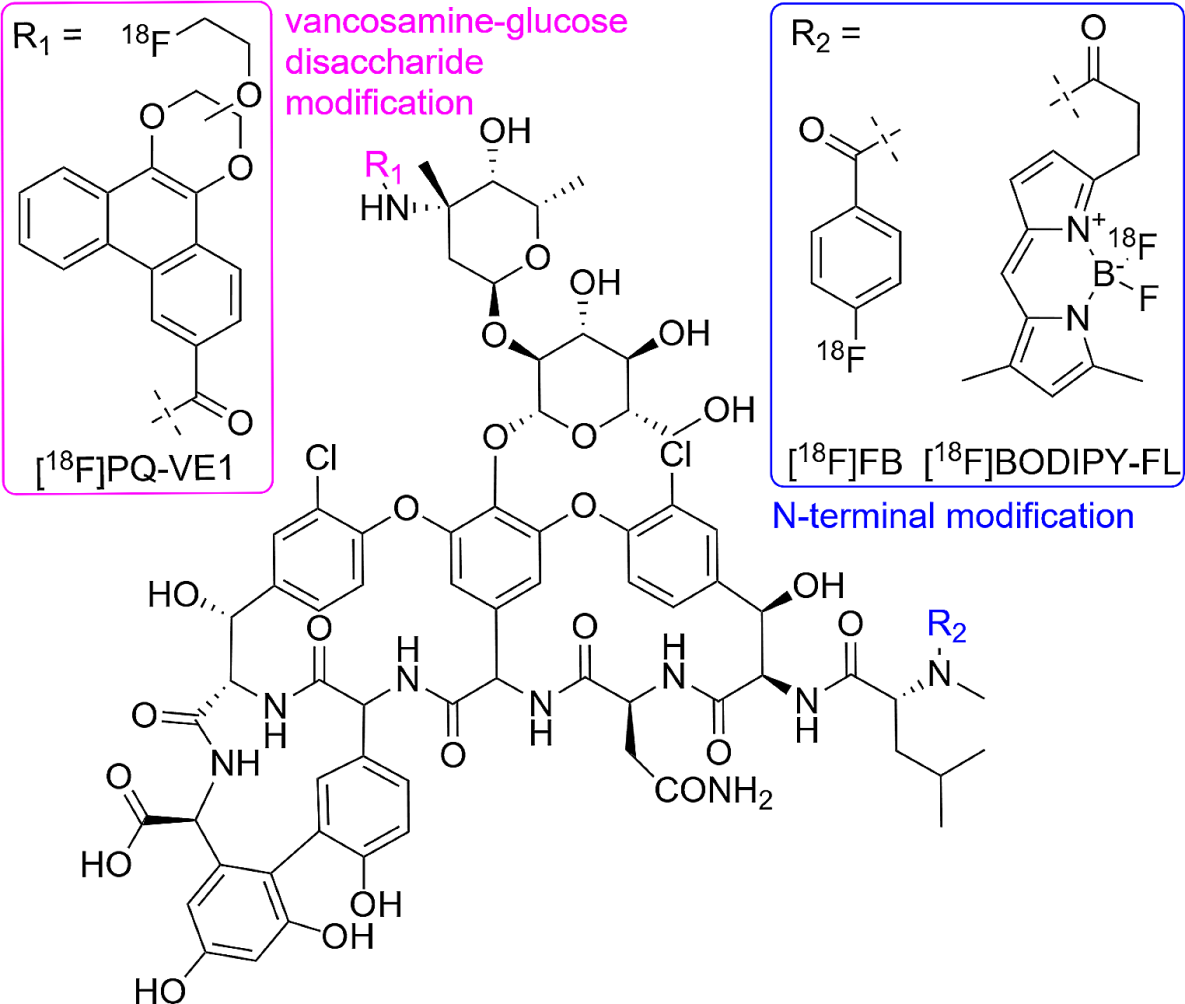
Chemical structures of vancomycin, [^18^F]PQ-VE1, [^18^F]FB and [^18^F]BODIPY-FL. The nucleophilic amine moieties in vancomycin are indicated as R_1_ and R_2_, for the primary and secondary amine, respectively

Vancomycin bears two nucleophilic amine moieties that can participate in the labeling reaction with an electrophilic ^18^F-labeling agent (Fig. 1) [44, 53, 54]. A primary amine is located on the vancosamine-glucose disaccharide (indicated with R_1_), whilst the secondary amine is located in the peptide backbone (indicated R_2_). It was previously determined that [^18^F]PQ-VE1 is conjugated to the primary amine [44] under the used reaction conditions. In contrast, we found [^18^F]FB and [^18^F]BODIPY-FL to be conjugated to the secondary amine, whilst the other isomer was not observed in the MS spectra (Supplemental Figs. 4 & 5).

### Tracer Stability

Tracer stability was assessed in both PBS and human plasma. In PBS, over 90% of the [^18^F]FB-vancomycin remained stable for up to 120 min, whereas no intact [^18^F]FB-vancomycin was detectable after 40 min incubation in plasma. In contrast, > 90% of the [^18^F]BODIPY-FL-vancomycin remained stable in PBS and plasma for up to 120 min, as was also previously shown for [^18^F]PQ-VE1-vancomycin [44].

### Distribution Coefficient

To determine the lipophilicity of [^18^F]FB-vancomycin, [^18^F]BODIPY-FL-vancomycin and [^18^F]PQ-VE1-vancomycin, their distribution coefficients (LogD_7.4_) in *n*-octanol / PBS were determined (Table 2). All three vancomycin derivatives retained a negative LogD_7.4_. However, compared to the parent compound vancomycin (*in silico* predicted LogD_7.4_ -5.1 [55]), an increased LogD_7.4_ was measured for all derivatives, indicating decreased hydrophilicity.

**Table 2:** LogD_7.4_ values for vancomycin-based PET tracers

Tracer	LogD_7.4_
[^18^F]FB-vancomycin	-0.96 ± 0.03 (*n* = 12)
[^18^F]BODIPY-FL-vancomycin	-1.72 ± 0.02 (*n* = 10)
[^18^F]PQ-VE1-vancomycin	-0.51 ± 0.07 (*n* = 9)

### In vitro tracer binding to *S. aureus, E. coli* and other clinically relevant bacteria

**Fig. 2:**
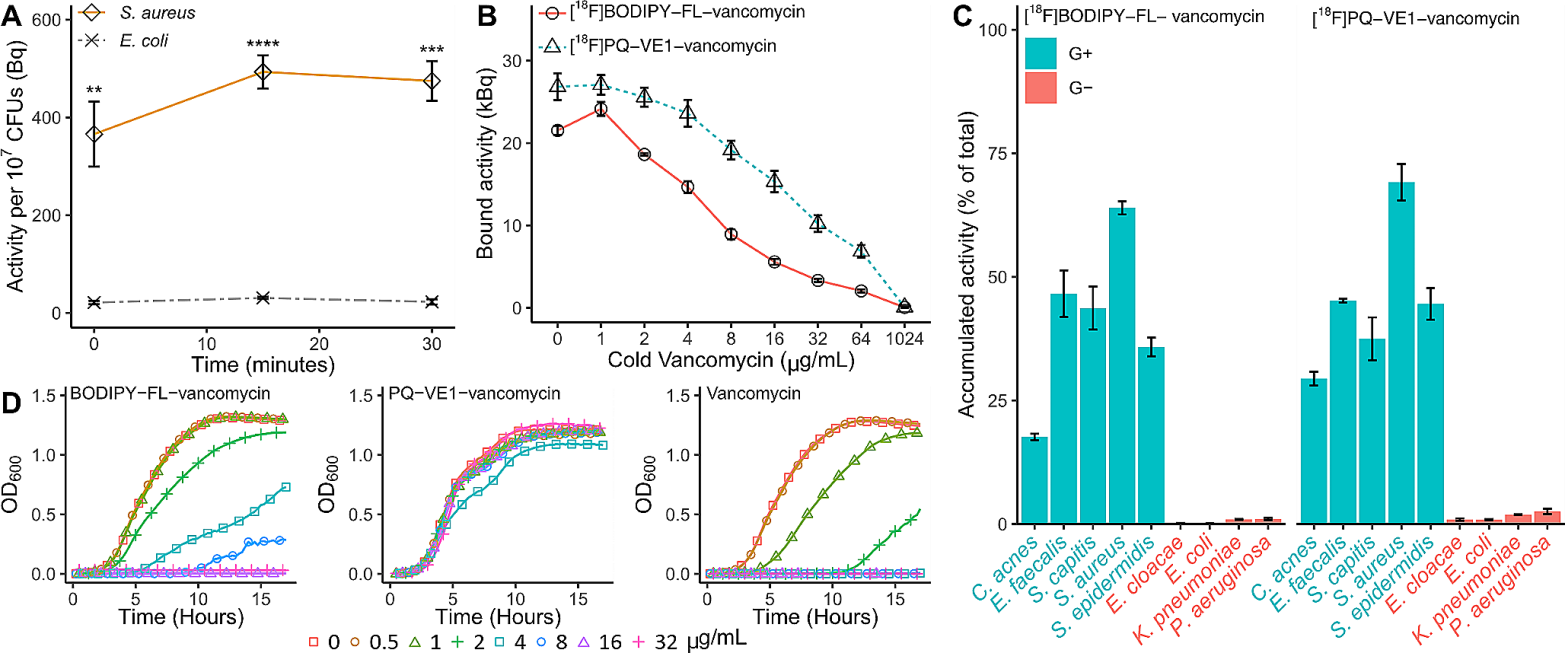
Tracer binding to staphylococci and other clinically relevant Gram-positive pathogens, and minimal inhibitory concentrations. **(A)** Incubation of Gram-positive (*S. aureus*) and Gram-negative (*E. coli*) bacteria with [^18^F]-BODIPY-FL-vancomycin shows selectivity towards the Gram-positive bacterium *S. aureus*. The plot shows the mean value ± SEM of the bound radioactivity per 10^7^ CFUs (** *p* < 0.01, *** *p* < 0.001, **** *p* < 0.0001). **(B)** Competition of unlabeled (‘cold’) vancomycin with [^18^F]PQ-VE1-vancomycin or [^18^F]BODIPY-FL-vancomycin for binding to *S. aureus* USA300. As more binding sites in the bacterial cell wall are occupied, a smaller fraction of PET tracer can bind to the bacteria. **(C)** A test panel of clinically relevant bacteria, including five Gram-positive (G+) bacterial species and four Gram-negative (G-) bacterial species, was incubated with tracer (left: [^18^F]BODIPY-FL-vancomycin, right: [^18^F]PQ-VE1-vancomycin). The results show selective tracer binding to Gram-positive bacterial species. **(D)** Conjugation of [^19^F]BODIPY-FL or [^19^F]PQ-VE1 to vancomycin results in an increased MIC, compared to the unmodified vancomycin

In vitro binding of [^18^F]BODIPY-FL-vancomycin and [^18^F]FB-vancomycin to the Gram-positive bacterium *S. aureus* and the Gram-negative bacterium *E. coli* was assayed by incubating the bacteria with either one of the two tracers and collecting samples at fixed time intervals to determine the radioactivity associated with the bacteria. For [^18^F]FB-vancomycin, no bacterial binding was observed, which is in line with the above observation that this tracer is highly unstable. In contrast, [^18^F]BODIPY-FL-vancomycin showed rapid and selective binding to *S. aureus*, but not to *E. coli* (Fig. 2A), as was previously reported for [^18^F]PQ-VE1-vancomycin [44].

To assess the specificity of the interaction of [^18^F]PQ-VE1-vancomycin or [^18^F]BODIPY-FL-vancomycin with *S. aureus*, a competition experiment was performed where each of these two tracers was incubated with the bacteria in the presence of increasing amounts of vancomycin. This showed that binding of [^18^F]PQ-VE1-vancomycin or [^18^F]BODIPY-FL-vancomycin is not significantly inhibited at vancomycin concentrations below 2 µg/mL (Fig. 2B). At vancomycin concentrations above 2 µg/mL, tracer binding to *S. aureus* is gradually reduced, but signal over background was still detected at vancomycin concentrations of up to 64 µg/mL. The binding of [^18^F]BODIPY-FL-vancomycin to *S. aureus* was outcompeted at slightly lower vancomycin concentrations than that of [^18^F]PQ-VE1-vancomycin, which is indicative of slight differences in tracer affinity for the staphylococcal cell wall. However, only at a concentration of 1024 µg/mL vancomycin the tracer binding was reduced to baseline.

To determine the extent of binding and specificity of [^18^F]BODIPY-FL-vancomycin and [^18^F]PQ-VE1-vancomycin to other bacteria, a panel of clinically relevant pathogens was assembled. Different strains of Gram-positive and Gram-negative bacteria were incubated with tracer, and the amount of bacteria-associated radioactivity was measured (Fig. 2C). The results show that the selectivity of [^18^F]BODIPY-FL-vancomycin towards Gram-positive bacteria, as presented in Fig. 2A, is not limited to *S. aureus*. Only minor differences in tracer accumulation were detected for different Gram-positive species, with the lowest values measured for *C. acnes*. None of the Gram-negative bacteria showed more than 1.0% and 2.6% accumulation for [^18^F]BODIPY-FL-vancomycin or [^18^F]PQ-VE1-vancomycin, respectively.

### Minimum Inhibitory Concentrations

To assess whether there is a risk that the usage of [^18^F]BODIPY-FL-vancomycin or [^18^F]PQ-VE1-vancomycin may elicit resistance to vancomycin, we compared the antibiotic activity of the reference standards of [^18^F]BODIPY-FL-vancomycin and [^18^F]PQ-VE1-vancomycin to that of unlabeled vancomycin. In particular, we determined the MIC of these tracers for the Gram-positive bacterium *S. epidermidis* ATCC 35,984 using ^19^F-fluorinated reference material. While the MIC of vancomycin for this bacterium was between 2.0 and 4.0 µg/mL with no residual growth at higher concentrations, the MIC of [^19^F]BODIPY-FL-vancomycin was increased to between 8.0 and 16 µg/mL (Fig. 2D). For [^19^F]PQ-VE1-vancomycin, the MIC value could not be determined, as none of the used concentrations resulted in growth inhibition of *S. epidermidis* (Fig. 2D). This implies that it is highly unlikely that clinical application of [^18^F]BODIPY-FL-vancomycin or [^18^F]PQ-VE1-vancomycin will elicit vancomycin resistance or favor the enrichment of vancomycin resistant bacteria in patients.

### Biodistribution in healthy animals and PET data

**Fig. 3:**
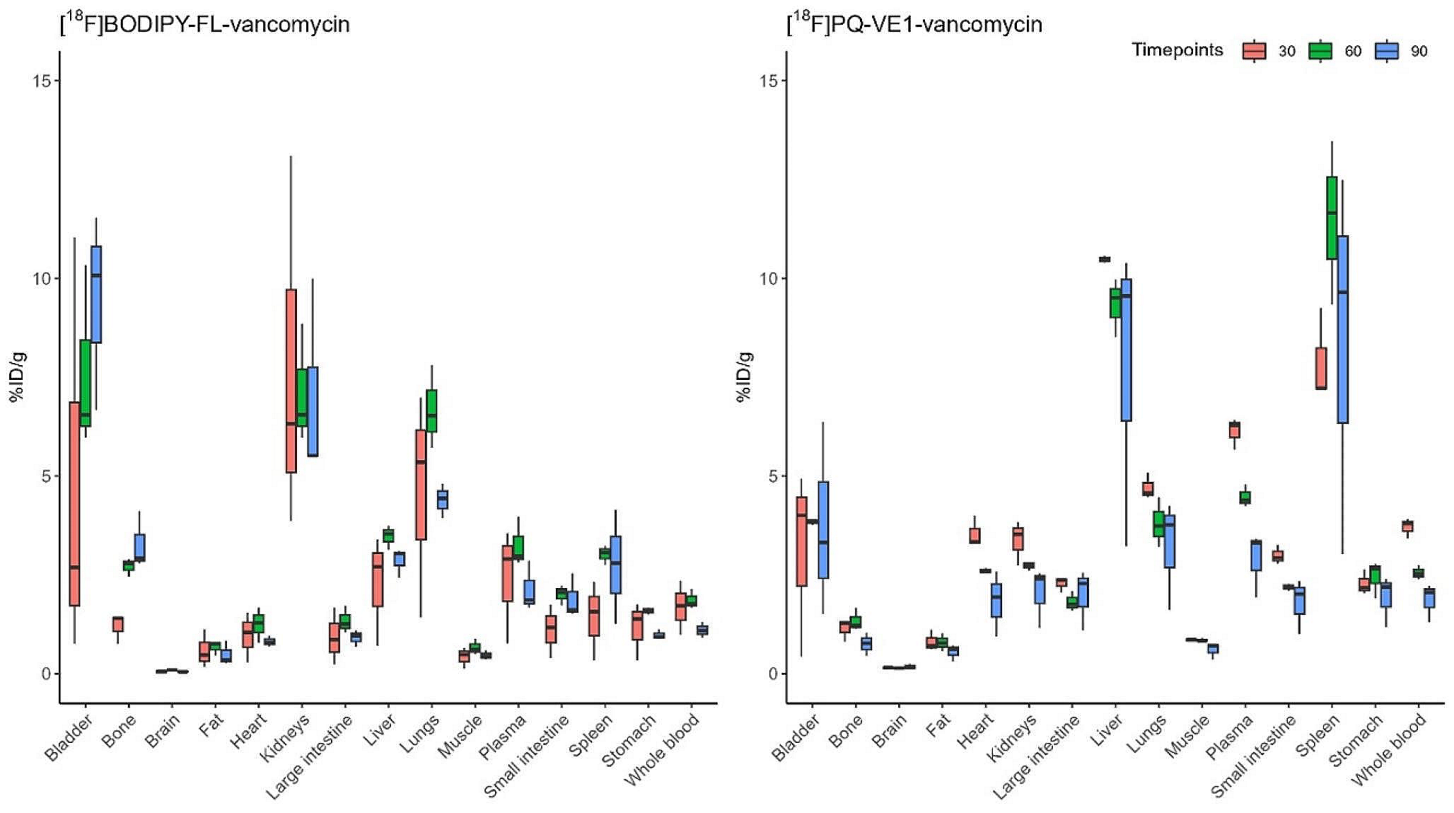
Biodistribution of [^18^F]BODIPY-FL-vancomycin (left) and [^18^F]PQ-VE1-vancomcin (right) in mice. Mice were injected with tracer and euthanized after 30, 60–90 min. Subsequently, organs and tissues were collected and accumulated radioactivity was measured with a gamma counter. Colors indicate the different groups (red: 30 min, green: 60 min, blue: 90 min). Data is expressed as percentage injected dose per gram (% ID/g), and represented as median ± interquartile range (IQR)

**Fig. 4:**
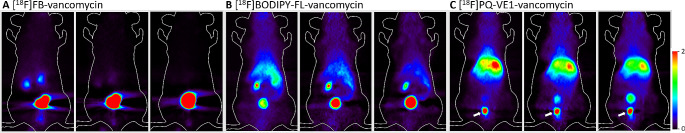
Representative PET images of mice injected with **(A)** [^18^F]FB-vancomycin, **(B)** [^18^F]BODIPY-FL-vancomycin or (**C**) [^18^F]PQ-VE1-vancomycin. Tracer was administered at t = 0 via penile vein injection (2.0 ± 0.9 MBq in 100 µL 0.9% saline solution). After tracer administration, animals were transferred to a microPET small animal scanner and dynamic data were recorded. The separated image frames show data recorded from 0–300 s, 900–1200 s and 2400–3000 s in A, and for 0–300 s, 900–1200 s and 3600–4800 s in B and C. A small subcutaneous depot (arrow) is visible below the bladder, as a result of the IV injection of [^18^F]PQ-VE1-vancomycin. All images are scaled to 0–2 standardized uptake value (SUV)

To determine tracer distribution in vivo, a biodistribution experiment was performed in healthy mice. Mice were injected intravenously with either [^18^F]FB-vancomycin, [^18^F]BODIPY-FL-vancomycin or [^18^F]PQ-VE1-vancomycin, and distribution of these tracers in different organs was determined using a calibrated gamma counter (Fig. 3). The injected mass was calculated to be 0.009 ng, 0.089 ng, and 0.159 ng, for [^18^F]PQ-VE1-vancomycin, [^18^F]FB-vancomycin and [^18^F]BODIPY-FL-vancomycin, respectively (Supplemental Table 2). As expected from the in vitro studies above, poor in vivo stability was observed for [^18^F]FB-vancomycin, as evidenced by rapid tracer accumulation in the bladder and urine (Supplemental Table 1). This was also observed by PET imaging, where some [^18^F]FB-vancomycin-derived signal was detectable in the kidneys during the first 300 s of the scan. However, thereafter most of the signal was detected in the bladder (Fig. 4A). Importantly, a completely different biodistribution pattern was observed for [^18^F]BODIPY-FL-vancomycin and [^18^F]PQ-VE1-vancomycin. The highest tracer signals were generally detected in well-vascularized organs, such as the liver, kidneys, and lungs. Some bone- and lung-uptake was detected for [^18^F]BODIPY-FL-vancomycin, which increased over time. There was also a substantial [^18^F]BODIPY-FL-vancomycin signal localized in the kidneys (Fig. 4B). For [^18^F]PQ-VE1-vancomycin, the predominant tracer-reservoir was the blood pool, indicating slower tissue uptake compared to [^18^F]BODIPY-FL-vancomycin (Fig. 4C). In line with published pharmacological data on vancomycin [56], the observed signals in the kidneys and bladder are indicative of renal tracer clearance. Interestingly, the spleen displayed the highest signal for [^18^F]PQ-VE1-vancomycin, whilst no elevated accumulation of [^18^F]BODIPY-FL-vancomycin was observed in this organ. Furthermore, neither [^18^F]BODIPY-FL-vancomycin nor [^18^F]PQ-VE1-vancomycin crossed the blood-brain barrier (BBB), as no substantial signal was observed in the brain. Importantly, uptake of both tracers in muscle tissue was low, which is a prerequisite for their use in the image-guided diagnosis of soft tissue infections, PJIs and FRIs.

## Discussion

In the present study, we set out to develop bacteria-selective PET imaging tracers using vancomycin as a targeting agent. Three different ^18^F-labelled tracers, namely [^18^F]FB-vancomycin, [^18^F]BODIPY-FL-vancomycin, and [^18^F]PQ-VE1-vancomycin, were synthesized and evaluated in vitro and in vivo (Table 3). Altogether, our analyses show that both [^18^F]BODIPY-FL-vancomycin and [^18^F]PQ-VE1-vancomycin bind specifically to Gram-positive bacteria in vitro and exhibit favorable biodistribution characteristics in vivo. In contrast, [^18^F]FB-vancomycin was rapidly degraded both in vitro and in vivo.

The synthesis of all three tracers can be performed on an Eckert & Ziegler Modular-Lab PharmTracer synthesis module within reasonable synthesis times and with A_m_ values ranging between 5 and 415 GBq/µmol. A limitation in the synthesis of [^18^F]BODIPY-FL-vancomycin is the inability to separate the ‘cold’ and ‘hot’ fractions after IEX due to their identical chemical structures. This results in a relatively low molar activity. Furthermore, direct IEX on BODIPY-FL-vancomycin did not yield any ^18^F-fluorinated product, which may be due to the presence of several acidic protons on vancomycin.

**Table 3:** Summary of the main characteristics of [^18^F]FB-vancomycin, [^18^F]BODIPY-FL-vancomycin and [^18^F]PQ-VE1-vancomycin

	Stability	Properties	Accumulation and clearance
[^18^F]FB-vancomycin	Rapidly degraded in vitro and in vivo.	LogD7.4 -0.96. Am 28.6 GBq/µmol.	Rapid renal clearance. No accumulation in tissues, only in urine.
[^18^F]BODIPY-FL-vancomycin	Stable in vitro and in vivo up to 2 h.	LogD7.4 -1.72. Am 5.35 GBq/µmol. MIC 16 µg/mL. Binding to Gram-positive bacteria in vitro.	Accumulation in liver, kidneys and lungs. Low muscle uptake.
[^18^F]PQ-VE1-vancomycin	Stable in vitro and in vivo up to 2 h.	LogD7.4 -0.51. Am 415 GBq/µmol. MIC > 32 µg/mL. Binding to Gram-positive bacteria in vitro.	Slow renal clearance. Accumulation in spleen, liver and lungs. Low muscle uptake.

Interestingly, two different conjugation sites in vancomycin were identified in the tracers. PQ was previously found to be conjugated to the primary amine of vancomycin [44], whilst we here describe conjugation of SFB and BODIPY-FL to the secondary amine of vancomycin. A possible explanation for these differences may be sought in the used conjugation conditions. This explanation is plausible, since Staroske et al. and Reessing et al. [53, 54]. reported differential reactivity between the two amines of vancomycin depending on the applied solvents, coupling reagents and linker-molecules. Importantly however, these structural differences in [^18^F]BODIPY-FL-vancomycin and [^18^F]PQ-VE1-vancomycin do not seem to impact their specific binding to Gram-positive bacteria like *S. aureus*. Our observations also show that conjugation to the primary or secondary amine of vancomycin is not a predictor for tracer stability as exemplified with [^18^F]PQ-VE1-vancomycin and [^18^F]BODIPY-FL-vancomycin, respectively.

Regarding the bacterial binding, comparable results were obtained for the previously developed [^18^F]PQ-VE1-vancomycin [44] and the presently developed [^18^F]BODIPY-FL-vancomycin. Both tracers were shown to bind effectively to all tested Gram-positive bacteria, but not to Gram-negative bacteria. On the other hand, the use of [^18^F]FB-vancomycin did not result in any bacterial binding. This is presumably related to its poor stability. Interestingly, much larger compounds have successfully been conjugated to vancomycin, such as BODIPY-FL and IRDye800CW, without impacting the bacterial binding [43, 53, 57]. Moreover, in vivo [^18^F]FB-vancomycin was rapidly degraded and excreted via the kidneys and bladder. At present, we do not know why [^18^F]FB-vancomycin is unstable in human plasma and in mice, but we presume that this relates to, as yet unidentified, enzymatic activity. This was not further investigated in view of the favorable tracer features presented by [^18^F]PQ-VE1-vancomycin and [^18^F]BODIPY-FL-vancomycin. Of note, the value of [^18^F]SFB in tracer development was previously demonstrated by its conjugation to IL2, as the resulting [^18^F]FB-IL2 conjugate was stable in human plasma at 37 °C [51, 58].

Antimicrobial activity of a compound is typically expressed as the MIC value, i.e., the lowest concentration of the compound at which no bacterial growth is detectable. The MIC of vancomycin is known to be influenced by chemical modifications [42, 59–61]. For the optical bacteria-targeted imaging agent vancomycin-IRDye800CW, it was shown that the conjugation of vancomycin with IRDye800CW led to an increased MIC value, without affecting binding of the conjugate to the target bacteria [53]. In our present study, a similar behavior of vancomycin conjugates was observed, as increased MICs were observed for [^18^F]BODIPY-FL-vancomycin and [^18^F]PQ-VE1-vancomycin. Nonetheless, both these tracers bind effectively and specifically to the target site of vancomycin in the bacterial cell wall, as underscored by the competition with unlabeled vancomycin. We consider the high MICs of [^18^F]BODIPY-FL-vancomycin and [^18^F]PQ-VE1-vancomycin as an advantage, because they are unlikely to provoke bacterial resistance when used as a PET tracer. This view is underscored by correlating the injected tracer mass to the clinical breakpoint of vancomycin for *S. aureus*, as the administered dose of [^18^F]BODIPY-FL-vancomycin is 1·10^4^-fold lower than the clinical breakpoint, and that of [^18^F]PQ-VE1-vancomycin is even 4·10^5^-fold lower. Importantly, our competition experiment with unlabeled vancomycin also shows that at clinically relevant serum levels of vancomycin of about 15 µg/mL [62], both [^18^F]BODIPY-FL-vancomycin and [^18^F]PQ-VE1-vancomycin will effectively bind to Gram-positive bacteria like *S. aureus*. This implies that [^18^F]BODIPY-FL-vancomycin and [^18^F]PQ-VE1-vancomycin, once approved for clinical implementation, can still be applied for the detection of Gram-positive bacterial infections even if a patient is already undergoing prophylactic treatment with vancomycin.

From the LogD_7.4_ data, it is clear that the conjugation of vancomycin with either one of the applied lipophilic prosthetic groups leads to a reduction in hydrophilicity compared to the unconjugated vancomycin. The increase in LogD_7.4_ is most pronounced for [^18^F]PQ-VE1-vancomycin. As high lipophilicity of drugs leads to reduced renal clearance rates [63, 64], it is not surprising that [^18^F]PQ-VE1-vancomycin exhibited slower clearance in mice than [^18^F]BODIPY-FL-vancomycin. However, the higher lipophilicity of [^18^F]PQ-VE1-vancomycin does not, apparently, have a negative influence on bacterial binding. This view is underscored by the competition experiment with the unlabeled vancomycin where [^18^F]BODIPY-FL-vancomycin was more readily outcompeted in bacterial binding than [^18^F]PQ-VE1-vancomycin. Importantly, in future experiments, it needs to be determined which rate of tracer clearance will be best-suited for the in vivo detection of bacterial infections at optimal target-to-background ratios. This is likely to depend on multiple factors, including not only the lipophilicity of the tracers, but also other factors like their route of administration and dose. In any case, based on the observed biodistribution in mice with low radioactivity uptake in muscle tissue, we consider the possibilities for in vivo application of [^18^F]BODIPY-FL-vancomycin and [^18^F]PQ-VE1-vancomycin to diagnose Gram-positive bacterial infections as highly promising, particularly when the extremities are involved as is the case in PJIs and FRIs. Overall, the main tracer reservoirs appear to be the well-vascularized organs, which is in accordance with existing literature for vancomycin [40, 56, 65]. Furthermore, little tracer accumulation was observed in brain, bone, fat and muscle tissue. The lack of tracer accumulation in the brain may be attributed to the hydrophilicity of both [^18^F]PQ-VE1-vancomycin and [^18^F]BODIPY-FL-vancomycin as reflected by their LogD_7.4_, because a more lipophilic nature gives drugs a higher propensity to cross the blood-brain-barrier (BBB) [66]. Importantly, the low tracer accumulation in uninfected bone, fat and muscle tissue is a favorable feature for vancomycin-based PET tracers, as high a-specific tracer accumulation would complicate accurate identification of Gram-positive pathogens at these preferred niches for bacterial infection.

## Conclusion

Here we present three different vancomycin-based tracers for the diagnosis of Gram-positive bacterial infections. Despite being based on the same targeting backbone, stark differences in stability and biodistribution of these tracers were observed depending on the applied prosthetic group. Given the effective in vitro binding to Gram-positive bacteria as exemplified for MRSA with [^18^F]BODIPY-FL-vancomycin and [^18^F]PQ-VE1-vancomycin, as well as the observed slow renal clearance and the low non-specific muscle, fat, and bone accumulation in mice, we consider vancomycin-based bacteria-targeted PET imaging as a promising application for the diagnosis of Gram-positive bacterial soft tissue and implant-associated infections. It is the objective of our ongoing studies to evaluate the performance of [^18^F]BODIPY-FL-vancomycin and [^18^F]PQ-VE1-vancomycin in preclinical in vivo models for bacterial infection.

### Electronic supplementary material

Below is the link to the electronic supplementary material.

Supplementary Material 1

## Data Availability

The datasets used and/or analyzed during the current study are available from the corresponding author on reasonable request.
