# Supplementary material for: Synthesis and preclinical evaluation of novel 18F-vancomycin-based tracers for the detection of bacterial infections using positron emission tomography
Source: Eur J Nucl Med Mol Imaging. 2024 Apr 22;51(9):2583–96. doi: 10.1007/s00259-024-06717-7 (PMC11224109; doi:10.1007/s00259-024-06717-7)
Supplement: Supplementary file 1 — Supplementary Material 1 [file 259_2024_6717_MOESM1_ESM.docx]

## Supplemental Figure 1. Eckert & Ziegler Modular-Lab PharmTracer synthesis module


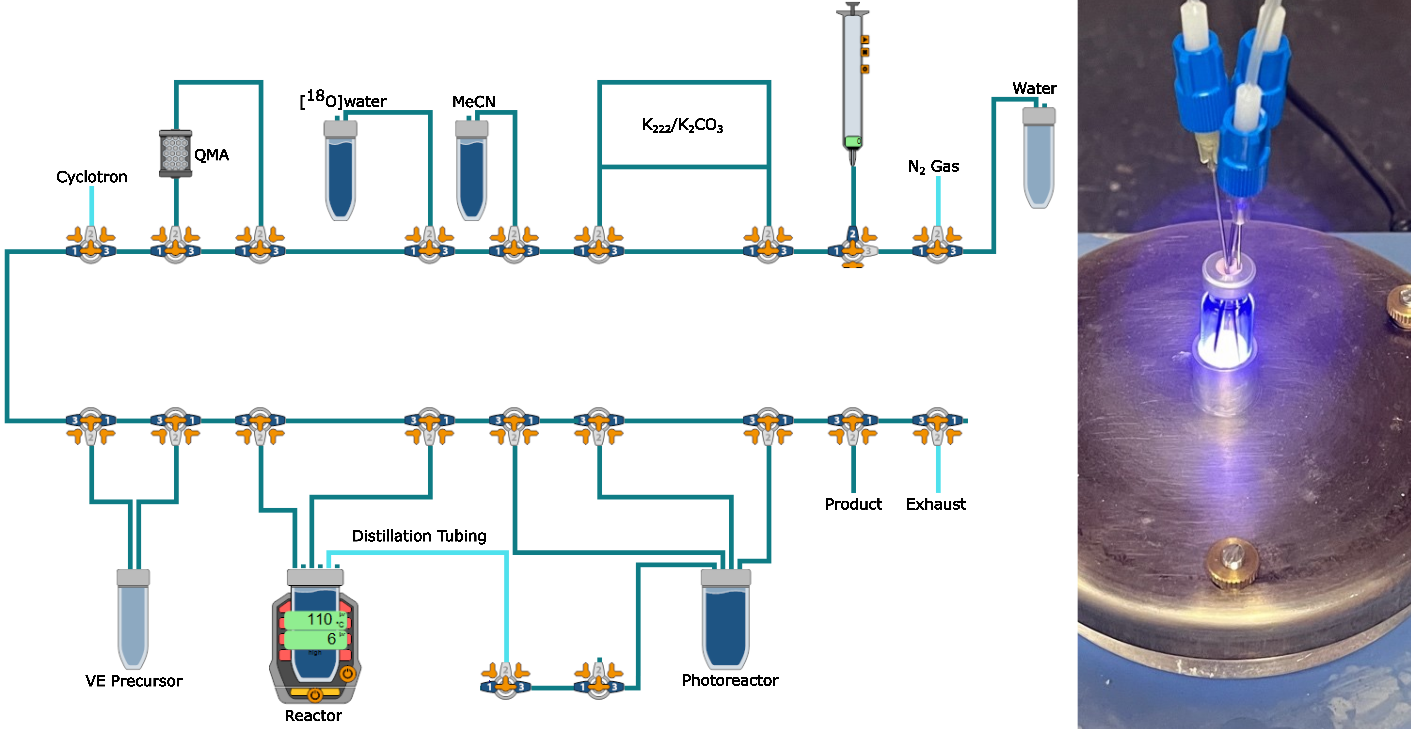
**Supplemental figure 1**. Schematic representation of the Eckert & Ziegler Modular-Lab PharmTracer synthesis module and photoreactor. In the photoreactor, a 2 mL borosilicate vial, containing 3 mg PQ-vancomycin in water/acetonitrile, is placed on top of a 10 W LED cob with appropriate driver. After distillation of [^18^F]VE1, the photoreactor is activated for 300 seconds. The photograph shows the activated LED reactor with vented 2 mL borosilicate vial and connecting lines.

## Supplemental Figure 2. HPLC chromatograms of [^18^F]FB-vancomycin


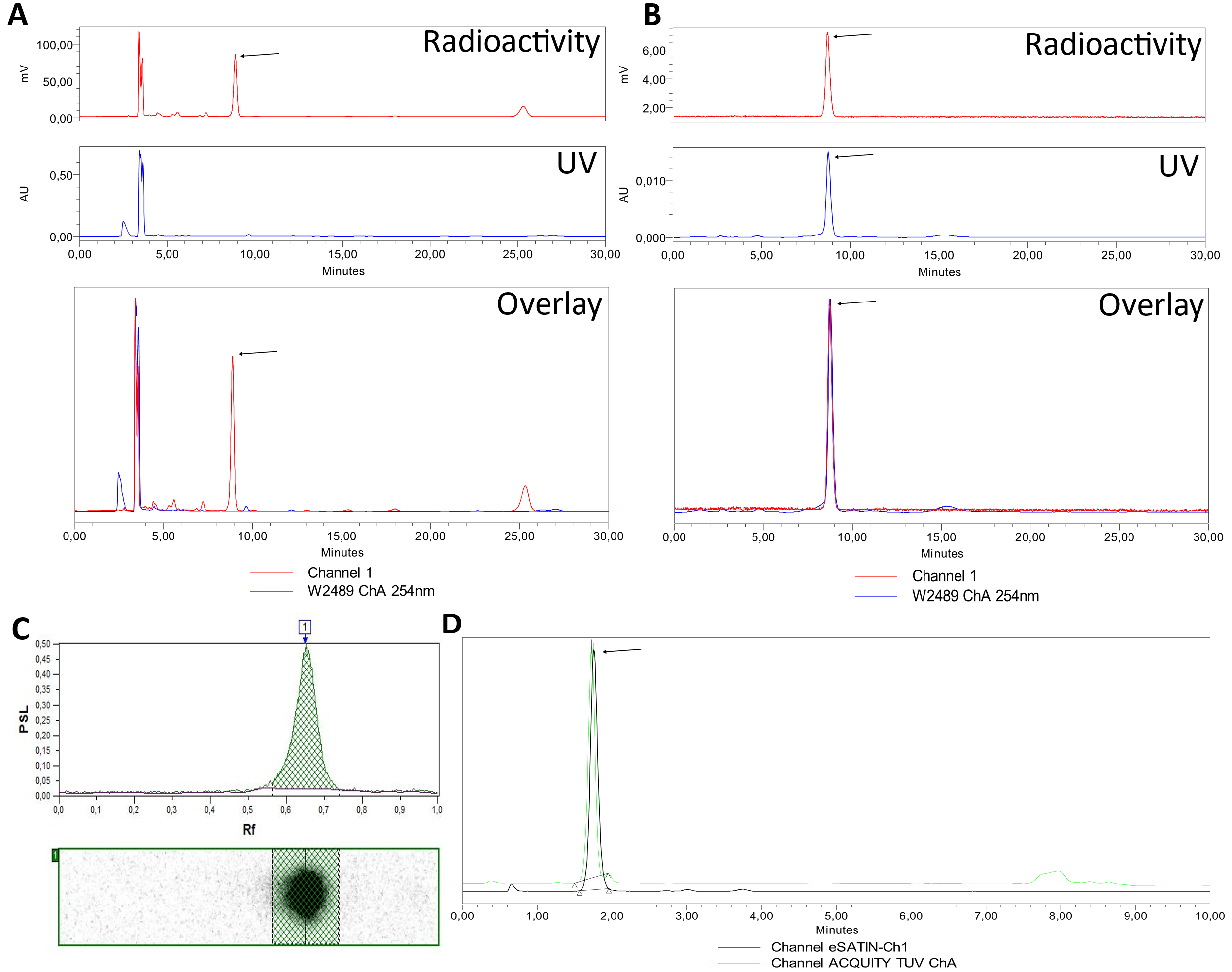


**Supplemental figure 2**. (A) Representative HPLC chromatogram for the crude reaction mixture of [^18^F]FB-vancomycin, with a retention time of 9 min for [^18^F]FB-vancomycin (indicated with arrow), and (B) collected product, spiked with cold [^19^F]FB-vancomycin. (C) Representative radio-TLC chromatogram of purified [^18^F]FB-vancomycin. (D) Representative UPLC chromatogram. Purity of the produced tracer was independently assessed by UPLC, using an independent analytical method (column: Acquity UPLC BEH Shield RP18 1.7 µm, solvent A: 100mM ammonium acetate in water, solvent B: MeCN, gradient 0 min: 95 % A, 4 min: 95 % A, 8 min: 70 % A, 10 min: 95 % A, flow: 0.8 mL · min^-1^, retention time 1.8 min).

## Supplemental Figure 3. HPLC chromatograms of [^18^F]BODIPY-FL-vancomycin


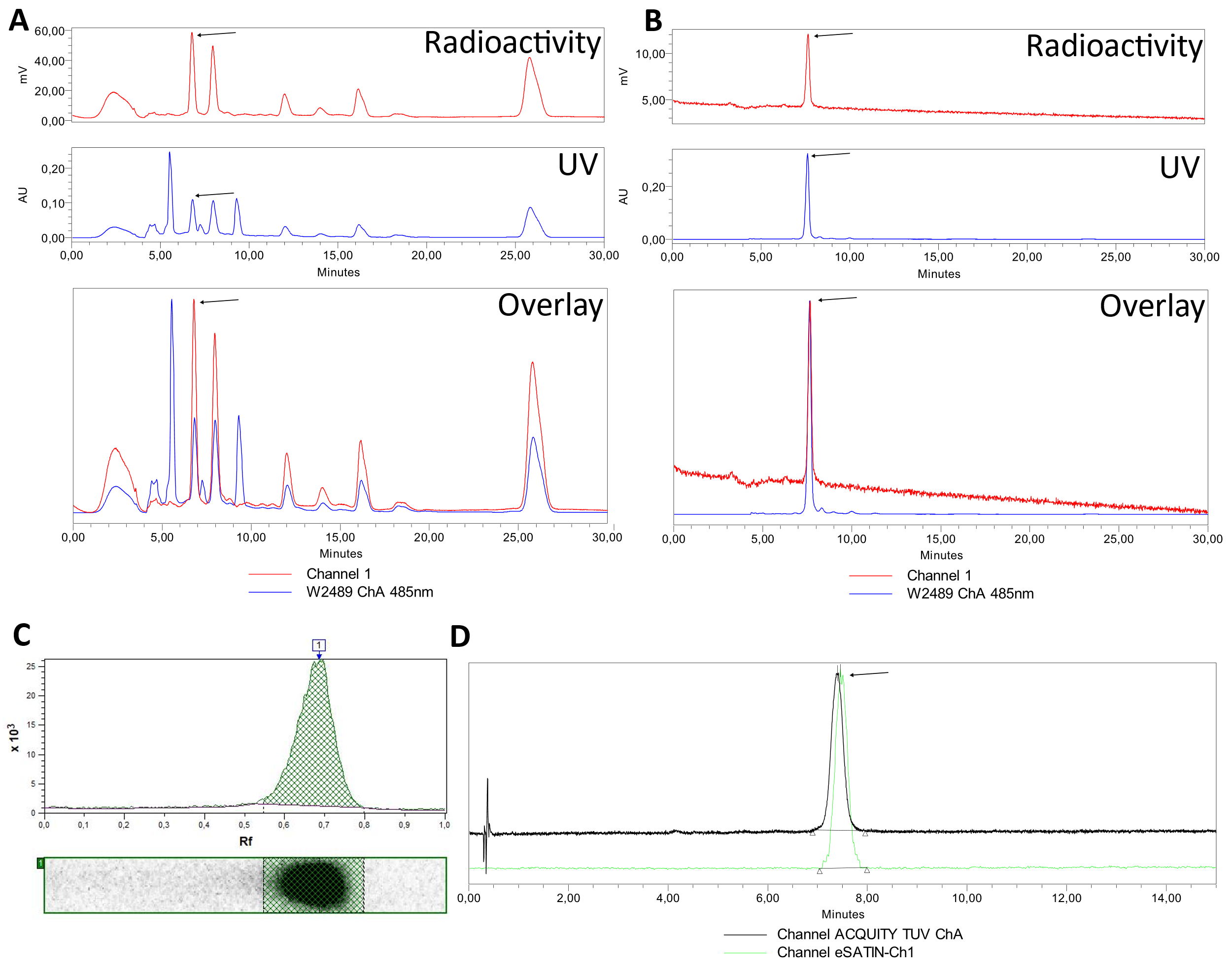


**Supplemental figure 3**. (A) Representative HPLC chromatogram for the crude reaction mixture of [^18^F]BODIPY-FL-vancomycin, with a retention time of 7 min for [^18^F]BODIPY-FL-vancomycin (indicated with arrow) and (B) collected product, spiked with cold [^19^F]BODIPY-FL-vancomycin. (C) Representative radio-TLC chromatogram of purified [^18^F]BODIPY-FL-vancomycin. (D) Representative UPLC chromatogram. Purity of the produced tracer was independently assessed by UPLC, using an independent analytical method (column: Acquity UPLC BEH Shield RP18 1.7 µm, solvent A: 80% water (0.1 % TFA), solvent B: 20 % MeCN (0.1 % TFA), flow: 0.8 mL · min^-1^, retention time 7.6 min).

Supplemental figure 4. MS [^19^F]FB-vancomycin


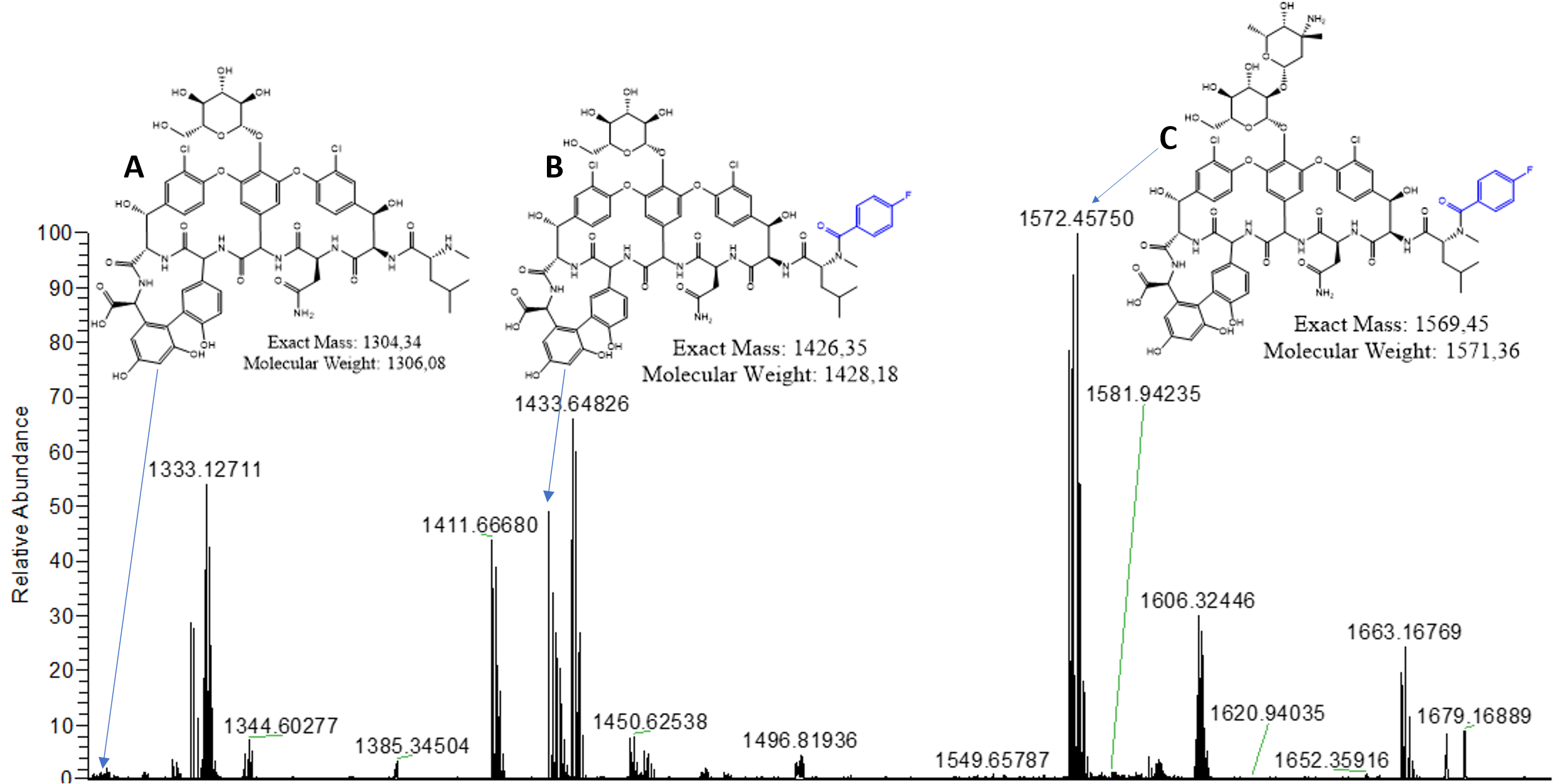


**Supplemental figure 4**. MS spectrum of [^19^F]FB-vancomycin. In order to determine if [^19^F]FB is conjugated to the primary amine or secondary amine of vancomycin, peaks can be identified that correspond to the full structure, or partial fragments thereof. One such fragment involves the loss of vancosamine, which also bears the primary amine. Depending on the conjugation site, the mass of the [^19^F]FB-vancomycin conjugate fragment will shift 122 Da. Potential fragments, representing the loss of a vancosamine moiety and conjugate (indicated with A) or vancosamine moiety (indicated with B) are shown in insets, together with the full structure (indicated with C). No appreciable signal is observed for the species with [^19^F]FB conjugated to the primary amine at 1304 Da (A). Instead, a peak from vancomycin with [^19^F]FB conjugated to the secondary amine is visible at 1426 Da (B).

## Supplemental Figure 5. MS [^19^F]BODIPY-FL-vancomycin


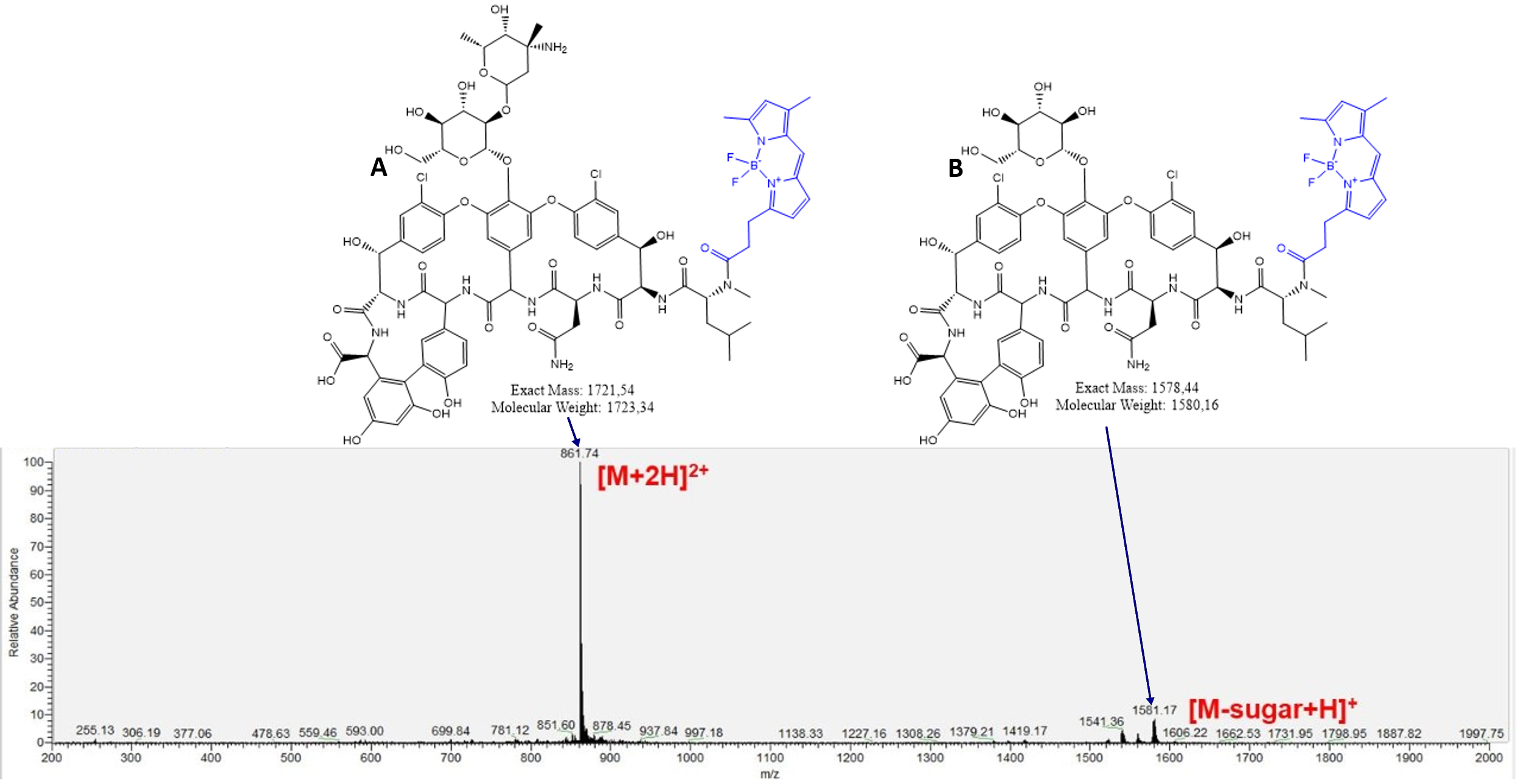


**Supplemental figure 5**. MS spectrum of BODIPY-FL-vancomycin. In order to determine if [^19^F]BODIPY-FL is conjugated to the primary amine or secondary amine of vancomycin, peaks can be identified that correspond to the full structure, or partial fragments thereof. One such fragment involves the loss of vancosamine, which also bears the primary amine. Depending on the conjugation site, the mass of the [^19^F]BODIPY-FL-vancomycin conjugate fragment will shift. The full structure (indicated with A) is shown as [M+2H]^2+^, together with a potential fragment representing the loss of a vancosamine moiety (indicated with B) are shown in insets. No signal is observed corresponding to the species with [^19^F]BODIPY-FL conjugated to the primary amine. Instead, a peak from vancomycin with [^19^F]BODIPY-FL conjugated to the secondary amine is shown as [M-sugar+H]^+^.

## Supplemental Table 1. Murine biodistribution data

Collected biodistribution data per time point. Mean ± SD.

|  | [^18^F]BODIPY-FL-vancomycin | | | | | | [^18^F]PQ-VE1-vancomycin | | | | | | [^18^F]FB-vancomycin | | | | | |
| --- | --- | --- | --- | --- | --- | --- | --- | --- | --- | --- | --- | --- | --- | --- | --- | --- | --- | --- |
|  | 30 min | | 60 min | | 90 min | | 30 min | | 60 min | | 90 min | | 30 min | | 60 min | | 90 min | |
| Organ | %ID/g | SD | %ID/g | SD | %ID/g | SD | %ID/g | SD | %ID/g | SD | %ID/g | SD | %ID/g | SD | %ID/g | SD | %ID/g | SD |
| Bladder | 4.83 | 5.46 | 7.62 | 2.37 | 9.43 | 2.50 | 3.12 | 2.38 | 3.83 | 0.06 | 3.73 | 2.46 | 21.75 | 8.05 | 18.52 | 9.53 | - | - |
| Bone | 1.20 | 0.39 | 2.71 | 0.23 | 3.27 | 0.73 | 1.14 | 0.29 | 1.34 | 0.28 | 0.75 | 0.29 | 0.06 | 0.02 | 0.04 | 0.02 | - | - |
| Brain | 0.05 | 0.03 | 0.09 | 0.01 | 0.05 | 0.01 | 0.14 | 0.02 | 0.14 | 0.01 | 0.17 | 0.07 | 0.05 | 0.00 | 0.05 | 0.00 | - | - |
| Fat | 0.59 | 0.48 | 0.67 | 0.18 | 0.49 | 0.31 | 0.81 | 0.26 | 0.79 | 0.23 | 0.55 | 0.21 | 0.54 | 0.35 | 0.26 | 0.05 | - | - |
| Heart | 0.96 | 0.64 | 1.25 | 0.45 | 0.81 | 0.13 | 3.54 | 0.40 | 2.60 | 0.07 | 1.82 | 0.83 | 0.11 | 0.02 | 0.22 | 0.18 | - | - |
| Kidneys | 7.77 | 4.79 | 7.13 | 1.53 | 7.00 | 2.59 | 3.37 | 0.56 | 2.73 | 0.11 | 2.04 | 0.76 | 1.01 | 0.28 | 0.45 | 0.18 | - | - |
| Large intestine | 0.93 | 0.72 | 1.34 | 0.35 | 0.91 | 0.21 | 2.28 | 0.19 | 1.81 | 0.26 | 1.98 | 0.77 | 0.25 | 0.05 | 0.37 | 0.24 | - | - |
| Liver | 2.27 | 1.40 | 3.47 | 0.31 | 2.86 | 0.37 | 10.48 | 0.09 | 9.33 | 0.74 | 7.72 | 3.92 | 0.15 | 0.03 | 0.10 | 0.01 | - | - |
| Lungs | 4.59 | 2.86 | 6.68 | 1.06 | 4.39 | 0.44 | 4.71 | 0.33 | 3.80 | 0.64 | 3.21 | 1.40 | 0.20 | 0.00 | 0.14 | 0.08 | - | - |
| Muscle | 0.42 | 0.27 | 0.66 | 0.20 | 0.46 | 0.12 | 0.86 | 0.03 | 0.85 | 0.04 | 0.60 | 0.22 | 0.14 | 0.12 | 0.12 | 0.08 | - | - |
| Plasma | 2.41 | 1.46 | 3.25 | 0.63 | 2.13 | 0.64 | 6.12 | 0.40 | 4.47 | 0.29 | 2.88 | 0.83 | 0.29 | 0.02 | 0.15 | 0.05 | - | - |
| Small intestine | 1.10 | 0.68 | 2.01 | 0.26 | 1.89 | 0.56 | 2.99 | 0.25 | 2.19 | 0.09 | 1.79 | 0.70 | 0.30 | 0.12 | 0.19 | 0.05 | - | - |
| Spleen | 1.41 | 1.00 | 3.02 | 0.25 | 2.73 | 1.44 | 7.89 | 1.18 | 11.49 | 2.07 | 8.39 | 4.85 | 0.14 | 0.05 | 0.09 | 0.03 | - | - |
| Stomach | 1.16 | 0.73 | 1.59 | 0.09 | 0.98 | 0.13 | 2.28 | 0.32 | 2.45 | 0.47 | 1.92 | 0.65 | 0.10 | 0.01 | 0.21 | 0.24 | - | - |
| Urine | 86.45 | 49.01 | 71.94 | 18.62 | 88.11 | 10.47 | 16.59 | 7.45 | 8.70 | 2.45 | 15.23 | 6.27 | 68.87 | 20.71 | 78.25 | 45.67 | - | - |
| Whole blood | 1.68 | 0.69 | 1.86 | 0.25 | 1.10 | 0.20 | 3.71 | 0.25 | 2.56 | 0.18 | 1.86 | 0.49 | 0.20 | 0.04 | 0.10 | 0.02 | - | - |

Biodistribution tables for all tracers. As limited *in vivo* stability was observed for [^18^F]FB-vancomycin, no experiment was performed in which mice injected with this tracer were monitored over a time course of 90 min. %ID/g: percentage injected dose per gram; SD: standard deviation.

## Supplemental Table 2. Injected masses per tracer in murine tracer biodistribution experiments

|  | [^18^F]BODIPY-FL-vancomycin | [^18^F]PQ-VE1-vancomycin | [^18^F]FB-vancomycin |  |
| --- | --- | --- | --- | --- |
| Molar Activity | 5.35 ± 3.91 | 415 ± 210 | 28.6 ± 14.5 | GBq/µmol |
| Molecular Weight | 1723 | 1788 | 1579 | Da |
| Injected Activity | 1.05 ± 0.30 | 2.13 ± 0.23 | 2.82 ± 0.40 | MBq |
| Blood volume^*^ | 2 | 2 | 2 | mL |
| Injected Mass | 0.338 ± 0.096 | 0.009 ± 0.001 | 0.156 ± 0.022 | ng |
| Mass/L | 0.169 | 0.00459 | 0.078 | µg/L |

^*^Assuming a murine blood volume of 79 mg/kg, 2 mL was used in the calculations for mice weighing 25.
